# Beyond the Skin: Nail Clues to a Bone Disease

**DOI:** 10.7759/cureus.99174

**Published:** 2025-12-14

**Authors:** Mariana Costa, Catarina Tavares, Cristina Alves, Paulo Coelho, João Nascimento

**Affiliations:** 1 Pediatrics Department, Coimbra Local Health Unit, Coimbra, PRT; 2 Pediatrics Department, Viseu Dão-Lafões Local Health Unit, Viseu, PRT; 3 Pediatric Orthopedics Department, Coimbra Local Health Unit, Coimbra, PRT; 4 Medical Imaging Department, Coimbra Local Health Unit, Coimbra, PRT; 5 Pediatrics Department - Rheumatology Unit, Coimbra Local Health Unit, Coimbra, PRT

**Keywords:** autoinflammatory bone disease, chronic recurrent multifocal osteomyelitis, pediatric rheumatology, psoriasis, trachyonychia

## Abstract

Chronic recurrent multifocal osteomyelitis (CRMO) is a rare autoinflammatory bone disorder that predominantly affects children, and approximately 25% patients also present with an associated inflammatory condition. Most patients respond to nonsteroidal anti-inflammatory drugs, but refractory cases may need corticosteroids, bisphosphonate therapy, or disease-modifying antirheumatic drugs (DMARDs) like methotrexate, sulfasalazine, tumor necrosis factor-alpha (TNF-α) inhibitors, or even anti-interleukin-1 (anti-IL-1) blockers. We report the case of a previously healthy nine-year-old girl presenting with a four-week history of right hip pain and limping, associated with recent swelling and pain over the left sternoclavicular joint. There was no fever or systemic symptoms. Laboratory tests showed elevated inflammatory markers, with negative antinuclear antibody and HLA-B27. Multifocal inflammatory bone lesions involving the right femoral neck, left clavicle, left proximal tibia, and right pubic symphysis were identified on imaging, consistent with CRMO. Bone biopsy excluded malignancy. Physical examination showed dystrophic changes in all fingernails, with a history of nail pitting for one year, leading to the diagnosis of CRMO associated with psoriasis. Treatment with naproxen, topical calcitriol, and methotrexate resulted in complete clinical remission and resolution of nail dystrophy within eighteen months. This case highlights the importance of recognizing CRMO as part of the psoriatic disease spectrum in children presenting with multifocal bone pain and nail changes. As reported in the literature, CRMO is often associated with other inflammatory disorders, such as psoriasis, with an estimated prevalence of around 4%. Moreover, a thorough physical examination was essential in this case, allowing the identification of trachyonychia or twenty-nail dystrophy, a potential manifestation of conditions such as alopecia areata, lichen planus, or psoriasis, which may precede cutaneous lesions and, in 1-5% of patients, may be the only manifestation of the disease, making its diagnosis particularly challenging.

## Introduction

Chronic recurrent multifocal osteomyelitis (CRMO) is a rare autoinflammatory bone disorder, with an annual incidence of 0.4-2.3 per 100,000 children [1], and peak onset between seven and fourteen years of age [2,3]. It is characterized by recurrent episodes of bone pain associated with sterile inflammatory lesions [2,4], most frequently involving the metaphyses of long bones, but the axial skeleton and clavicle may also be affected [3,4,5]. Systemic features such as low-grade fever or malaise may occur, though many children remain otherwise well [3].

CRMO is increasingly recognized as part of a broader spectrum of chronic non-infectious osteitis and can overlap clinically with juvenile spondyloarthritis (SpA), although classical SpA features - such as male predominance, uveitis, or urethritis - are typically absent [6]. Up to one-quarter of patients present with an associated inflammatory condition, most commonly psoriasis, palmoplantar pustulosis, or inflammatory bowel disease [3,7]. Nail psoriasis, in particular, may precede cutaneous lesions and may be the sole manifestation of psoriatic disease in a minority of patients.

Genetic susceptibility plays a role in CRMO, with nearly half of first- and second-degree relatives exhibiting associated inflammatory conditions, most commonly psoriasis or inflammatory bowel disease [3,7,8]. Both complex multifactorial inheritance and rare monogenic forms have been described, including Majeed syndrome (LPIN2 mutations) and DIRA syndrome (IL1RN mutations) [9]. In contrast, most sporadic cases likely arise from a polygenic or multifactorial background, and current evidence suggests that CRMO results from an imbalance between pro- and anti-inflammatory cytokines [6,9].

Diagnosis of CRMO is challenging and requires exclusion of infectious, malignant, and other inflammatory conditions. Should be based on a combination of clinical, histopathological, microbiological, and imaging findings [2-4]. Imaging plays a central role: radiographs may be normal or nonspecific, bone scintigraphy helps detect multifocal disease and asymptomatic foci [3,5,7] (although interpretation can be challenging due to physiologic uptake in growth plates [4,7]) and whole-body magnetic resonance imaging (MRI) is the most sensitive imaging modality for detecting active and asymptomatic lesions and is also valuable for monitoring disease activity and soft-tissue inflammation, while avoiding radiation exposure [3,4,6]. A biopsy is often performed when malignancy cannot be confidently excluded.

Management is largely empirical and guided by disease severity. Nonsteroidal anti-inflammatory drugs (NSAIDs) are considered first-line therapy, especially in patients without vertebral involvement, while corticosteroids, disease-modifying antirheumatic drugs (DMARDs), such as methotrexate or sulfasalazine, and biologic agents are used in refractory cases [2,3,6,9]. Recently, the 2025 European Alliance of Associations for Rheumatology (EULAR) and the American College of Rheumatology (ACR) classification criteria have provided a standardized framework to support diagnosis in clinical practice [1].

## Case presentation

A previously healthy nine-year-old girl presented with right hip pain lasting four weeks, associated with nocturnal awakenings and limping. She also reported pain and swelling over the left sternoclavicular region for approximately two to three weeks. There was no history of fever, anorexia, weight loss, night sweats, or other associated symptoms, and no recent trauma or infectious episode. The patient had no personal or family history of osteoarticular disorders or any other relevant medical conditions. Laboratory investigations revealed elevated inflammatory markers (C-reactive protein 5 mg/dL and erythrocyte sedimentation rate 61 mm/hour), with negative antinuclear antibody and HLA-B27.

**Figure 1:**
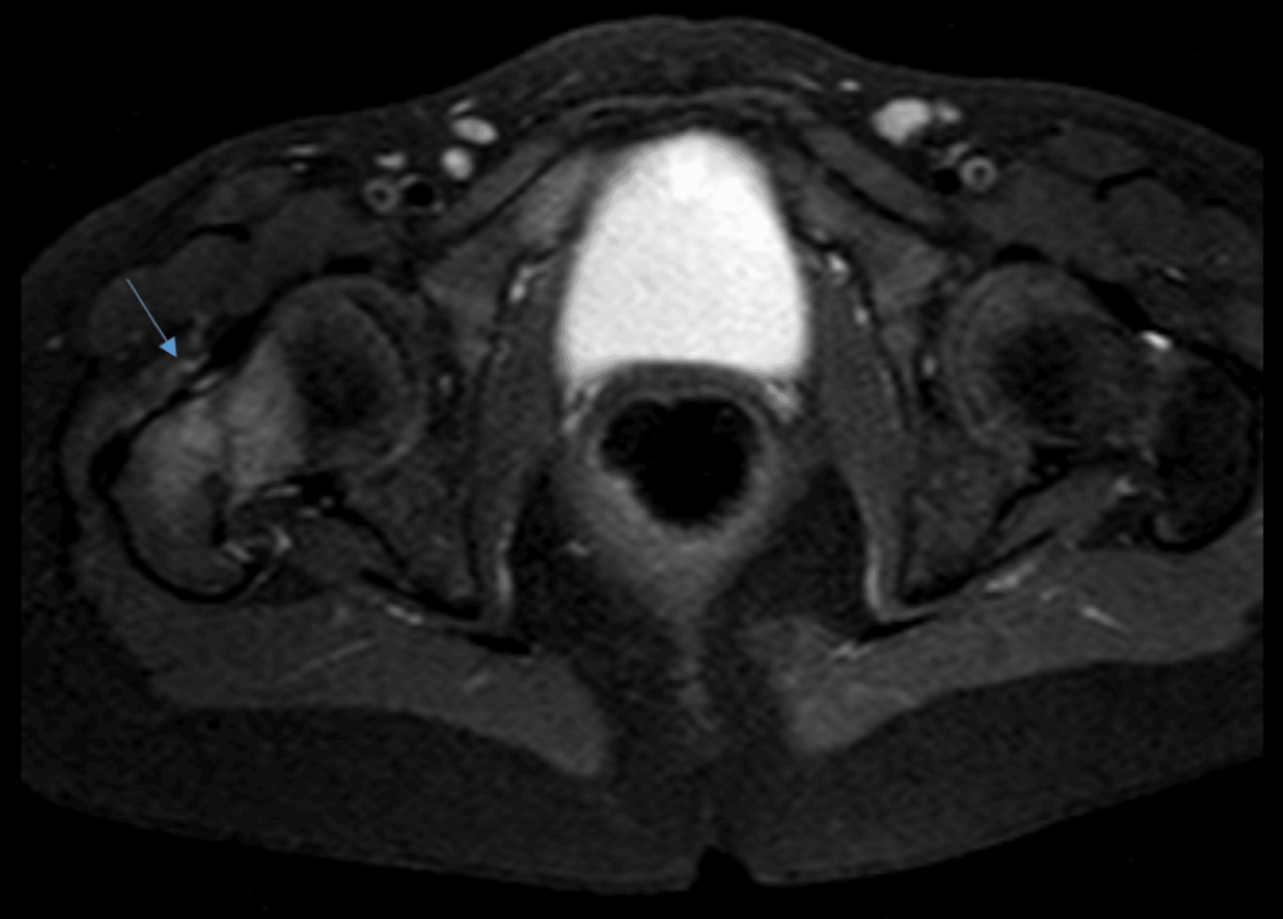
Right hip MRI showing area of edema involving the right femoral neck and the intertrochanteric region (blue arrow).

Ultrasound of the right hip joint was unremarkable; however, MRI demonstrated “an area of edema involving the right femoral neck and intertrochanteric region” (Figure 1). Ultrasound of the left sternoclavicular joint revealed “bony irregularity at the clavicular aspect of the left sternoclavicular joint, with joint effusion, synovial thickening, and increased echogenicity of adjacent fat planes, suggestive of inflammatory changes.

**Figure 2:**
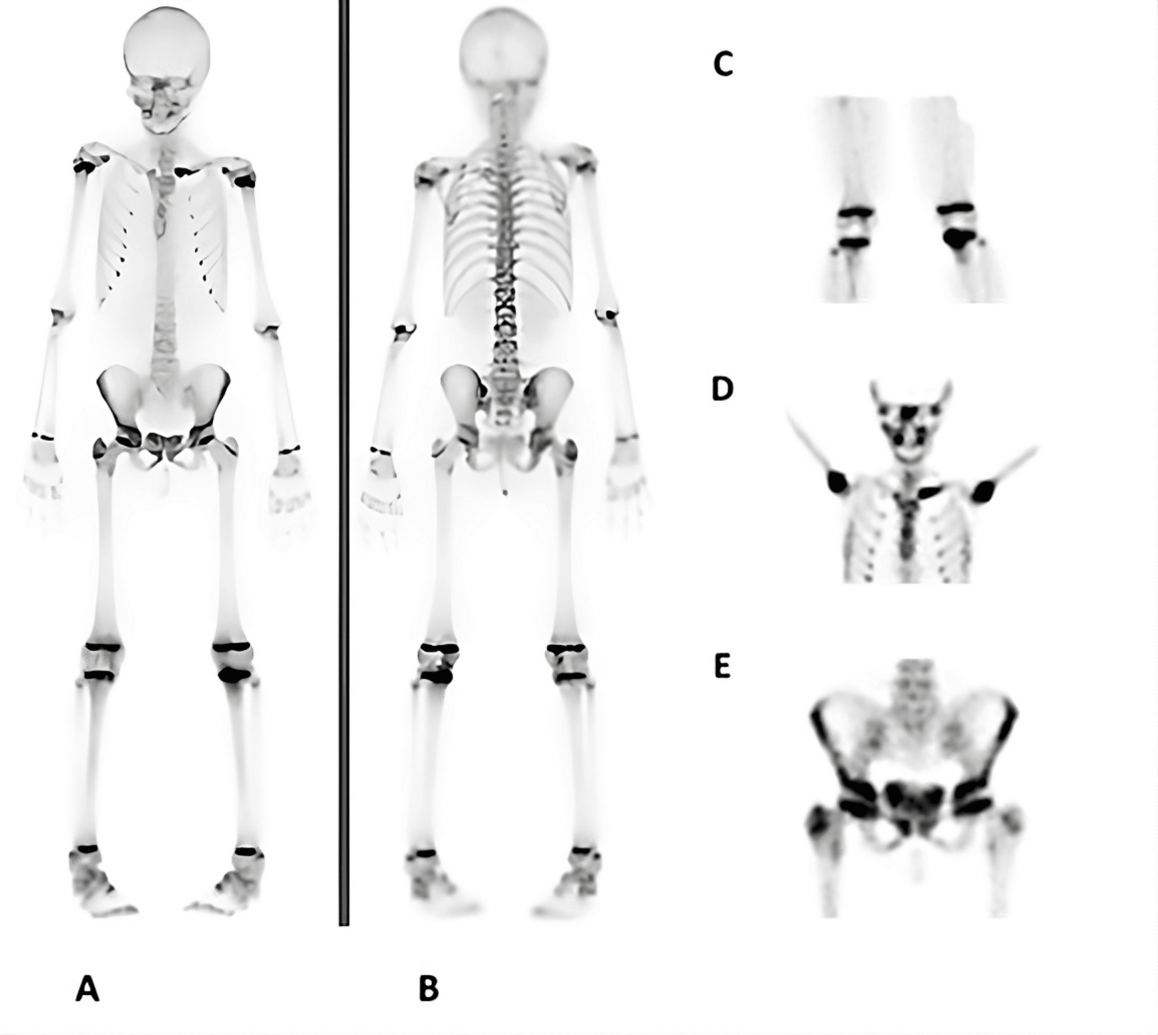
Bone scintigraphy. (A) Whole-body anterior view showing increased vascularization and radiotracer uptake in the right greater trochanter, femoral neck, and acetabular margin. (B) Whole-body posterior view. (C) Focal increased uptake in the left proximal tibia. (D) Increased uptake in the medial aspect of the left clavicle. (E) Increased uptake in the right pubic symphysis.

At orthopedic evaluation, she presented with swelling of the left sternoclavicular joint with tenderness on palpation, discomfort during right hip mobilization with limitation of abduction and both internal and external rotation, and an antalgic gait with right-sided limping. Bone scintigraphy revealed increased vascularization and radiotracer uptake in the right greater trochanter, femoral neck, and acetabular contour, as well as in the left proximal tibia, the inner aspect of the left clavicle, and the right pubic symphysis (Figure 2).

A bone biopsy of the femur ruled out sarcoma and myeloproliferative or lymphoproliferative disorders.

**Figure 3:**
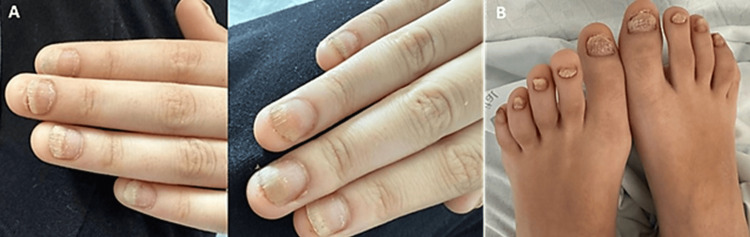
Hands (A) and toes (B) showing trachyonychia, involving all fingernails and toenails, characterized by rough, thin, and brittle nail plates (“twenty-nail dystrophy”).

Nail dystrophy in all 20 nails was identified only later in the course of follow-up, at which point her mother reported a one-year history of nail pitting (Figure 3).

The case was discussed in a multidisciplinary meeting due to the coexistence of nail lesions and multifocal bone involvement. Rheumatology evaluation confirmed the diagnosis of CRMO, in the probable context of psoriasis.

The patient has a score of 64, according to the new EULAR/ACR classification criteria. Naproxen therapy was initiated at a maximum dose of 250 mg twice daily (~15 mg/kg/day), achieving significant control of bone pain, and dermatology evaluation confirmed the diagnosis of psoriasis. The patient started topical calcitriol treatment, resulting in partial improvement of nail dystrophy.

At one-year follow-up, the patient experienced disease relapse, presenting with pain in the right upper limb, particularly around the elbow region, along with persistent, pronounced nail dystrophy. A new bone scintigraphy showed minimal to no significant increased radiotracer uptake at the right greater trochanter, the proximal left tibia (epiphyseal and adjacent metaphyseal regions), the right pubic symphysis, and medially at the left clavicle, compared with the previous study. No other areas of the skeleton show evidence of new focal bone lesions in the context of known chronic nonbacterial osteomyelitis, including the right upper limb. A whole-body MRI was not performed at that time, but it was considered as a potential follow-up imaging, in case the bone scintigraphy revealed new or progressive lesions.

Fecal calprotectin was also assessed, with a value of 83 mg/kg (considered near normal). Naproxen was reintroduced, and subcutaneous methotrexate was started at a maximum dose of 17.5 mg/week (~14.5 mg/m²/week), resulting in excellent clinical response and sustained improvement, including resolution of nail dystrophy, which only began to improve after methotrexate was started.

## Discussion

This case highlights the diagnostic challenges of CRMO, a rare autoinflammatory bone disorder in children that requires a high index of suspicion and a multidisciplinary approach [1,3,5]. CRMO typically presents with recurrent and remitting episodes of bone pain, typically worse at night, and may closely mimic bacterial osteomyelitis or malignant bone tumors, such as Ewing sarcoma or lymphoma [3,7,9]. Therefore, careful clinical assessment and appropriate imaging are essential for accurate diagnosis and to avoid unnecessary invasive procedures. In our patient, malignancy was appropriately excluded through bone biopsy, underscoring that CRMO remains a diagnosis of exclusion.

In response to the lack of standardized diagnostic definitions, the 2025 EULAR/ACR classification criteria provide a structured framework for diagnosing CRMO, requiring a total score of ≥55 out of 100 [1]. Our patient reached a score of 64 points, supporting the diagnostic certainty and emphasizing the usefulness of these criteria both in clinical practice and research [1].

Although most pediatric CRMO cases are sporadic and likely polygenic, rare monogenic IL-1-mediated disorders - such as DIRA (IL1RN mutations) and Majeed syndrome (LPIN2 mutations) - may present with CRMO-like phenotypes [7,9]. DIRA leads to uncontrolled IL-1 signaling and presents in infancy with systemic inflammation, severe multifocal osteomyelitis, and pustulosis, often fatal if untreated. Majeed syndrome is characterized by CRMO, congenital dyserythropoietic anemia, and neutrophilic dermatosis [7,9]. SAPHO syndrome (synovitis, acne, pustulosis, hyperostosis, and osteitis) also belongs to the spectrum of chronic non-infectious osteitis; although rare, with an estimated prevalence of <1:10,000 [6], its true frequency is likely underestimated due to variable presentations and overlapping nomenclature [4,6,9].

As reported in the literature, CRMO frequently coexists with other inflammatory conditions, most often psoriasis, as observed in our patient, palmoplantar pustulosis, or inflammatory bowel disease (most commonly Crohn’s disease) [3,5,7,10]. Increasing evidence supports the gut-bone and gut-skin axes as shared pathways linking the intestinal microbiota to skeletal and skin homeostasis through immune, metabolic, and inflammatory mechanisms. Dysbiosis can disrupt intestinal barrier integrity (“leaky gut”), promoting systemic inflammation through increased exposure to microbial products and heightened cytokine release (TNF-α, IL-6), which enhances osteoclastogenesis and promotes bone resorption. These immune and metabolic disturbances can also contribute to cutaneous inflammation, as seen in conditions like psoriasis and inflammatory bowel disease. In CRMO, such mechanisms likely converge, linking the gut-bone and gut-skin axes and influencing both disease activity and severity [10,11]. This helps explain the overlap between musculoskeletal and cutaneous inflammatory disorders observed in CRMO, psoriasis, and inflammatory bowel disease. These insights highlight the emerging role of the microbiome as a potential therapeutic and diagnostic target in chronic autoinflammatory bone diseases [11,12].

The primary goals of CRMO treatment are symptom control, preservation of bone growth and function, and prevention of deformity and recurrence [2,3,9,13]. NSAIDs are the first-line therapy for CRMO [1,2,3,6] and were initiated in this patient, providing initial improvement. Disease exacerbation later required the introduction of methotrexate, chosen over anti-TNF therapy due to the patient’s psoriatic phenotype, its efficacy in nail disease, favorable safety profile, and ease of administration.

In addition to corticosteroids, DMARDs and biologic agents, bisphosphonates such as pamidronate and zolendronate have also shown benefit in refractory cases, by reducing bone inflammation and promoting sustained remission. Pamidronate can be administered 1 mg/kg per dose (maximum 60 mg/dose), either monthly for at least three months or for three consecutive days every three months; a lower initial dose of 0.5 mg/kg may be used, and the annual cumulative dose should not exceed 11.5 mg/kg. Zoledronic acid is typically given at 0.0125-0.025 mg/kg every three to six months, with escalation to 0.05 mg/kg (maximum 4 mg/dose) based on disease activity and patient response. Close monitoring for acute-phase reactions and potential effects on bone mineral density is advised [13]. Due to potential adverse effects and limited evidence from controlled trials, biologic and bisphosphonate therapies are generally reserved for severe or treatment-resistant cases [2,3,10], particularly in patients refractory to NSAIDs or with active spinal lesions, which may lead to vertebral compression deformities, from mild anterior wedging to circumferential collapse (vertebra plana) [6].

Long-term follow-up is essential. Whole-body MRI is the most sensitive tool for evaluating disease burden and assessing treatment response, although its availability and need for sedation in young children may limit routine use. In such cases, a pragmatic combination of targeted MRI and bone scintigraphy can be adopted to guide follow-up [3,4,6].

We highlight the importance of a meticulous physical examination. In this patient, nail dystrophy became more evident during follow-up, prompting reassessment and ultimately supporting the diagnosis of CRMO associated with psoriasis. This case is distinctive due to the combination of multifocal CRMO, coexisting psoriasis, and trachyonychia - the latter serving as an early but subtle clue to the underlying autoinflammatory process. Trachyonychia, characterized by roughness of all nail plates, may precede or occur in the absence of cutaneous psoriasis and can represent the sole initial manifestation in up to 1-5% of patients [14]. Its recognition was essential, as nail changes in children are often overlooked, contributing to the delayed diagnosis of psoriatic disease. The coexistence of nail psoriasis and multifocal sterile osteitis provides further support for CRMO within the broader psoriatic disease spectrum.

## Conclusions

This case emphasizes the importance of integrating musculoskeletal and dermatologic findings when evaluating children with chronic bone pain. Recognition of subtle clinical clues, such as nail changes, can provide key insights into systemic inflammatory processes and facilitate early diagnosis and appropriate management of CRMO and its associated conditions. Early recognition and treatment, as well as multidisciplinary collaboration - particularly involving rheumatology, dermatology, and radiology - may help prevent bone deformity and long-term sequelae.
